# Impact of Low-Level-Viremia on HIV-1 Drug-Resistance Evolution among Antiretroviral Treated-Patients

**DOI:** 10.1371/journal.pone.0036673

**Published:** 2012-05-10

**Authors:** Constance Delaugerre, Sébastien Gallien, Philippe Flandre, Dominique Mathez, Rishma Amarsy, Samuel Ferret, Julie Timsit, Jean-Michel Molina, Pierre de Truchis

**Affiliations:** 1 Laboratoire de Virologie, Hôpital Saint Louis-APHP, Paris, France; 2 Université Paris 7 Paris Diderot, Paris, France; 3 INSERM U941, Paris, France; 4 Service de Maladies Infectieuses et Tropicales, Hôpital Saint Louis-APHP, Paris, France; 5 Université Pierre et Marie Curie, Paris, France; 6 INSERM U943, Paris, France; 7 Laboratoire Hématologie-Immunologie, Hôpital Raymond Poincaré-APHP, Garches, France; 8 Unité des MST, Hôpital Saint Louis-APHP, Paris, France; 9 Service de Maladies Infectieuses et Tropicales, Hôpital Raymond Poincaré-APHP, Garches, France; University of Pittsburgh, United States of America

## Abstract

**Background:**

Drug-resistance mutations (DRAM) are frequently selected in patients with virological failure defined as viral load (pVL) above 500 copies/ml (c/mL), but few resistance data are available at low-level viremia (LLV). **Our objective was** to determine the emergence and evolution of DRAM during LLV in HIV-1-infected patients while receiving antiretroviral therapy (ART).

**Methods:**

Retrospective analysis of patients presenting a LLV episode defined as pVL between 40 and 500 c/mL on at least 3 occasions during a 6-month period or longer while on the same ART. Resistance genotypic testing was performed at the onset and at the end of LLV period. Emerging DRAM was defined during LLV if never detected on baseline genotype or before.

**Results:**

48 patients including 4 naive and 44 pretreated (median 9 years) presented a LLV episode with a median duration of 11 months. Current ART included 2NRTI (94%), ritonavir-boosted PI (94%), NNRTI (23%), and/or raltegravir (19%). Median pVL during LLV was 134 c/mL. Successful resistance testing at both onset and end of the LLV episode were obtained for 37 patients (77%), among who 11 (30%) acquired at least 1 DRAM during the LLV period: for NRTI in 6, for NNRTI in 1, for PI in 4, and for raltegravir in 2. During the LLV period, number of drugs with genotypic resistance increased from a median of 4.5 to 6 drugs. Duration and pVL level of LLV episode, duration of previous ART, current and nadir CD4 count, number of baseline DRAM and GSS were not identified as predictive factors of resistance acquisition during LLV, probably due to limited number of patients.

**Conclusion:**

Persistent LLV episodes below 500 c/ml while receiving ART is associated with emerging DRAM for all drug classes and a decreasing in further therapeutic options, suggesting to earlier consider resistance monitoring and ART optimization in this setting.

## Introduction

HIV drug resistance is related to the selection of viral variants harbouring drug resistance associated mutations (DRAM) in the target genes of antiretroviral drugs, promoted by ongoing viral replication in patients receiving antiretroviral therapy (ART).

Cross-sectional studies showed that DRAMs occurred in 88% of HIV-infected patients on ART when virological failure (VF), defined as a plasma viral load (pVL) above 1000 copies/mL (c/mL) [1]. Moreover, accumulation of DRAMs increases when maintaining a failing drug regimen with pVL above 400 c/mL [2], [3], leading to a loss of future therapeutic options also due to a large cross-resistance between drug within each antiretroviral class. These data support the current guidelines which recommend a rapid therapeutic switch for a new potent regimen when VF is detected, including at least two fully active drugs.

In recent years, the improvement of assays to quantify pVL led to progressively decrease the threshold of VF. In French recommendations, VF was defined as two consecutive plasma HIV-1 RNA quantifications above 1000 copies/ml in 2004 [4], then above 500 copies/ml in 2006 [5]. Since 2008, VF is defined as a confirmed pVL above 50 c/mL in French and European guidelines [6], [7]. In addition, because of the current availability of several antiretroviral drugs targeting different steps of viral cycle, a larger proportion of HIV-infected patients are now receiving ART and most of them are virologically suppressed [8], [9], [10]. However some of them experienced persistent low level viremia (LLV) episodes, defined as repeated pVL between 50 and 500 or 1000 c/mL, under stable ART which, unlike intermittent viremia or *blip.* Episodes of LLV are reported associated with higher immune activation [11], increased risk of virologic failure [11], [12], and perhaps increased mortality [13].

Although accumulation of DRAM is well known for pVL above 400 c/mL, the diagnosis and the management of emerging drug resistance during LLV remain a clinical challenge since standard genotypic tests fail to amplify HIV-1 RNA below 500 c/mL in 45% [14] and conventional genotyping is recommended for pVL above 1,000 c/mL [15]. Hence without data of resistance genotyping test, during LLV, clinicians can either switch by excess to a new suppressive therapy when the virus is still sensitive or in contrary maintain the regimen when DRAM have already emerged and still accumulate.

To longitudinally detect the development of DRAM during LLV, we retrospectively analyzed HIV-1-infected patients followed in two French hospital clinical cohorts. We aim to describe the clinical and virological characteristics of those patients experiencing LLV, and to analyze the dynamics of emergence of genotypic drug-resistance in this setting.

## Methods

### Ethics statements

This study was a non-interventional study with no addition to usual procedures. Biological material and clinical data were obtained only for standard viral diagnostic following physicians' prescriptions (no specific sampling, no modification of the sampling protocol, no supplementary question in the national standardized questionnaire). Data analyses were carried out using an anonymized database. The protocol was approved by the local ethical research committee (Persons Protection Committee Ile-de-France XI, n°11062, july 2011), which confirmed exemption from patient informed consent, according to the French Public Health Law CSP Art. L 1121-1.1.

### Selection of patients

Subjects were retrospectively identified from a routine virological monitoring database from two French Infectious Diseases clinics. LLV cases were defined as subjects who experienced pVL between 50 and 500 c/mL on at least 3 occasions during a 6-month period or longer while on the same ART regimen. During the LLV period, no more than 3 pVL below 50 c/mL were allowed.

The onset of LLV period was different according to the pVL at the beginning of the current treatment. In naïve patients and in patients failing previous treatment, the onset of LLV period was defined as the first date of pVL between 50–500 c/mL during the follow-up after having received at least 6 months of the same ART regimen. In patients virologically suppressed, the onset of LLV period was defined as the date of the first pVL between 50–500 c/mL. The LLV period ended at the last low-level viral load measurement between 50–500 c/mL and before VF (defined as a confirmed pVL above 500 c/mL or a single pVL above 1000 c/mL), or before ART modification decided by the physician.

### Genotype analysis

To analyze the emergence of DRAM, resistance genotyping tests (RGT) were performed from several plasma time-points of the LLV period. The first RGT (G1) was performed at the beginning of the ART regimen under which LLV was detected, or at the onset of the LLV period. A second RGT (G2) was performed at the end of the LLV period or if the PCR amplification failed, from prior available samples with detectable viremia during the LLV period.

RNA was extracted from 1 to 1.5 ml of frozen plasma using the QIAamp viral RNA minikit (Qiagen, Courtaboeuf, France) after a 2 hours-ultracentrifugation at 28 000XG at 4°C to pellet virus particles. Regions of the *pol* gene containing protease, reverse transcriptase (RT) and integrase (for patients receiving raltegravir-containing regimen) genes were amplified by RT-PCR followed by nested PCR (www.hivfrenchresistance.org).

Population sequencing was performed on purified amplicons (ExoSap-IT Purification Kit, Abbott, Rungis, France) with the Taq Dye Deoxy Terminator cycle sequencing kits (Applied Biosytems, France) and resolved on an ABI 3730 automated DNA sequencer. Sequences were processed using SmartGene® HIV-1 Sequence Analysis and Database Module (SmartGene GmbH, Zug, Switzerland) and aligned to the HIV-1 subtype B reference strain HXB2 (GenBank accession no. K03455).

Drug-resistance mutations were identified according to the 2009 International AIDS Society (IAS)-USA list (www.iasusa.org) and drug susceptibility was assessed using the 2009 ANRS HIV-1 drug-resistance algorithm v18 (www.hivfrenchresistance.org). Genotypic susceptibility score (GSS) to the current regimen was calculated as the sum of active ( = 1), partially active ( = 0.5) and inactive ( = 0) drugs. Mixtures of a mutated and wild-type HXB2 strain were counted as mutations. Mutations present at G1 were assumed to be still present at G2 as it has been shown that once a mutation has been accumulated, it persists in minority viruses even if it is not detected by population sequencing [16]. All DRAM selected during LLV period was considered as new one if never described in G1 or in any available RGT performed previously to the study.

Patients in whom RT or protease amplification failed were excluded from the resistance analysis.

### Statistical analysis

Descriptive statistics are given as median and Inter Quartil Range (IQR) for continuous variables and percent for categorical variables. A logistic regression model was used to investigate whether the following variables were associated with an increase risk of the emergence of a new DRAM: ART type, treatment duration, nadir and current CD4 cell count baseline pVL, number of DRAM at G1, GSS, duration of LLV period, level of pVL during LLV and number of LLV episodes above 50 c/mL.

## Results

### Baseline characteristics of patients

We included in the study 48 HIV-1-infected patients with an identified episode of LLV (Table 1). Patients were male (71%), with a median age of 44 years. They were HIV-1-infected since a median of 12 years. Patients had a median nadir CD4 cell count of 65 cells/mL and 28 patients (58%) were classified as CDC stage C. Four patients were receiving a first ART regimen and 44 were already ART-treated since a median time of 9 years. Those pretreated patients have received before the study a median number of nucleoside reverse transcriptase inhibitors (NRTI) of five, of non-nucleoside reverse transcriptase inhibitors (NNRTI) of one and of protease inhibitors (PI) of two.

**Table 1:** Baseline characteristics of 48 patients experiencing low-level viremia episodes.

		Total N = 48
Age (years)	Median (IQR)	44 (40–51)
Sex	Males, n (%)	34 (71%)
Time since HIV diagnosis (years)	Median (IQR)	12 (7–18)
CDC classification	Status C, n (%)	28 (58%)
Nadir CD4 (cells/ml)	Median (IQR)	65 (11–217)
HIV-1 subtype	Subtype B, n (%)	25 (52 %)
Duration of ART at baseline (years)	Median (IQR)	8 (4–10)
Previous antiretroviral drugs	NRTI, n (%)	5 (0–7)
	NNRTI, n (%)	1 (0–3)
	PI, n (%)	2 (0–7)
Patients with previous virological failure	n (%)	44 (92%)
CD4 at the onset of current ART	Median (IQR)	255 (98–430)
pVL at the onset of current ART	Median (IQR)	3.7 (2.1–4.7)

ART: antiretroviral therapy, NRTI: nucleoside reverse transcriptase inhibitors, NNRTI: non-nucleoside reverse transcriptase inhibitors, PI: protease inhibitors.

The median of pVL and CD4 cell count at the initiation of the current ART were 3.7 (Inter Quartil Range, 2.1 to 4.7) log10 c/mL and 255 (98 to 430) cells/mL, respectively. Current ART included at least 2 NRTI in 45 patients (94%): lamivudine (3TC) or emtricitabine (FTC) in 43 patients (90%), tenofovir (TDF) in 27 patients (56%) and abacavir (ABC) in 13 patients (27%). Forty five patients (94%) received ritonavir boosted-PI (PI/r): lopinavir (LPV) in 13 patients (29%) and darunavir (DRV) in 15 patients (33%). Eleven (23%) and 9 (19%) patients received NNRTI and raltegravir (RAL) based-regimen, respectively.

### Description of the LLV period

The median duration of the LLV period was 11 (9 to 16) months. During the LLV period, the median higher pVL was 222 (150 to 341) c/mL and the median of the average pVL was 134 (104 to 194) c/mL. The median number of pVL measurements above 50 c/mL was 4 (3 to 6). A single pVL above 500 c/mL was observed in 4 (8%) patients. The median CD4 cells count at entry in the LLV period was 371 [238 to 548] and the end of the LLV period the median was 415 [243 to 576].

### HIV drug resistance mutations before LLV period

**Figure 1:**
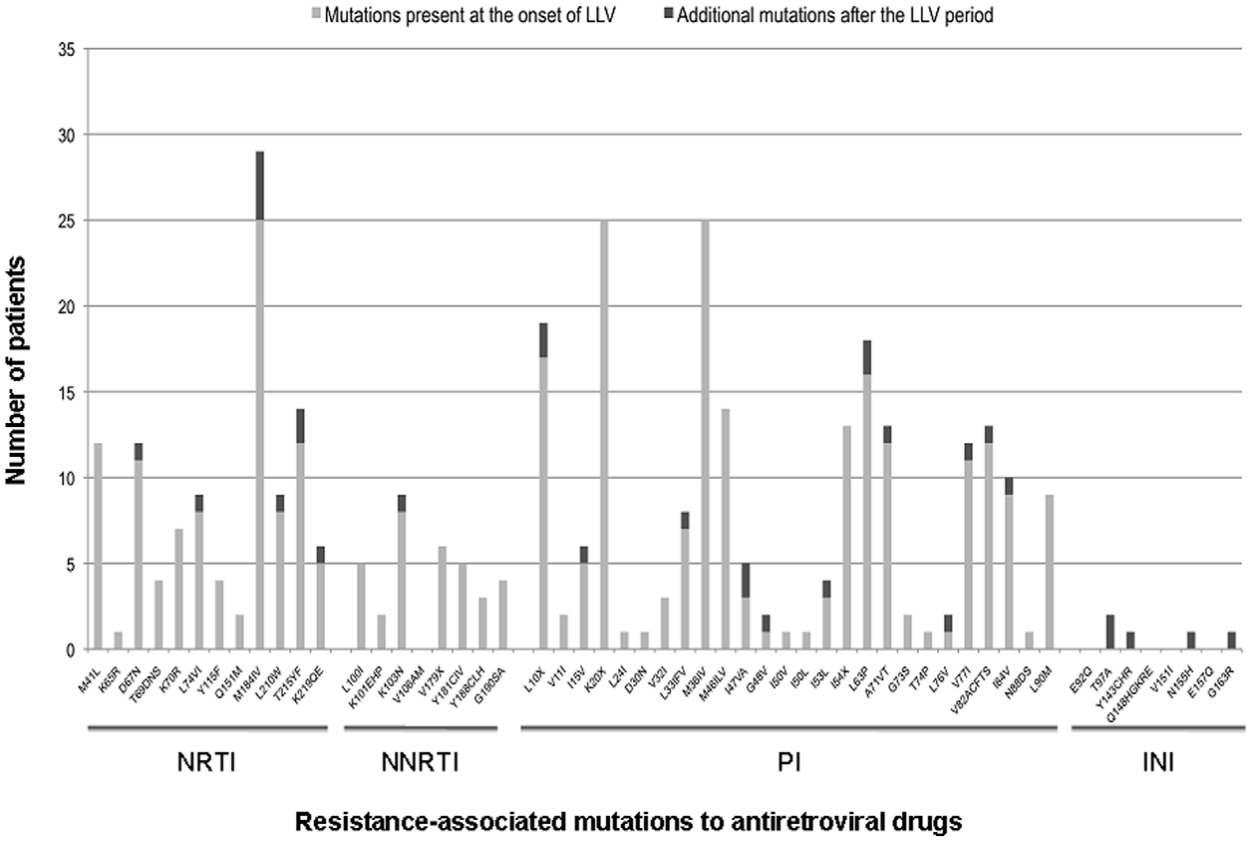
Resistance-associated mutations to nucleoside reverse transcriptase inhibitors (NRTI), non-nucleoside reverse transcriptase inhibitors (NNRTI), protease inhibitors (PI) and integrase inhibitors (INI) before and after the low-level viremia (LLV) period, according to the 2009 International AIDS Society-USA list.

For the 48 patients, we generated a total of 96 RT and PR sequences from the samples corresponding to G1 and G2, including 72 plasmas with low-level of replication (median 133 c/mL) for which the rate of successful amplification was 71% (51/72). For the 9 patients receiving a RAL-containing regimen, we successfully amplified the integrase gene in 9 from the 13 (75%) plasmas analyzed (median 213 c/mL). Overall, successful resistance genotypic tests for both G1 and G2 were obtained in 37 patients among 48 (77%) (Figure 1): three were receiving a first ART and 34 were already pretreated with at least one previous experience of VF. Among the 34 pretreated patients, the prevalence of drug resistance at the onset of LLV was assessed using only one RGT (G1) for 12 (35%) patients, two RGT (G1 and one previous test) for 8 (24%) and 3 or more RGT for 14 (41%).

The median (IQR) numbers of DRAM in G1 for each antiretroviral drug class were 3 (0 to 5) for NRTI, 1 (0 to 2) for NNRTI, 1 (0 to 4) for PI (major mutations) (Figure 1). The most frequent DRAM (≥20%) were found in the RT gene at codons 41 (32%), 67 (30%), 74 (22%), 184 (59%), 210 (22%), 215 (32%) and 103 (22%), and in the protease gene (major mutations) at codons 46 (38%), 54 (35%), 82 (32%), 84 (24%) and 90 (24%) (Figure 1). No DRAM was detected in the integrase gene before receiving RAL.

**Figure 2:**
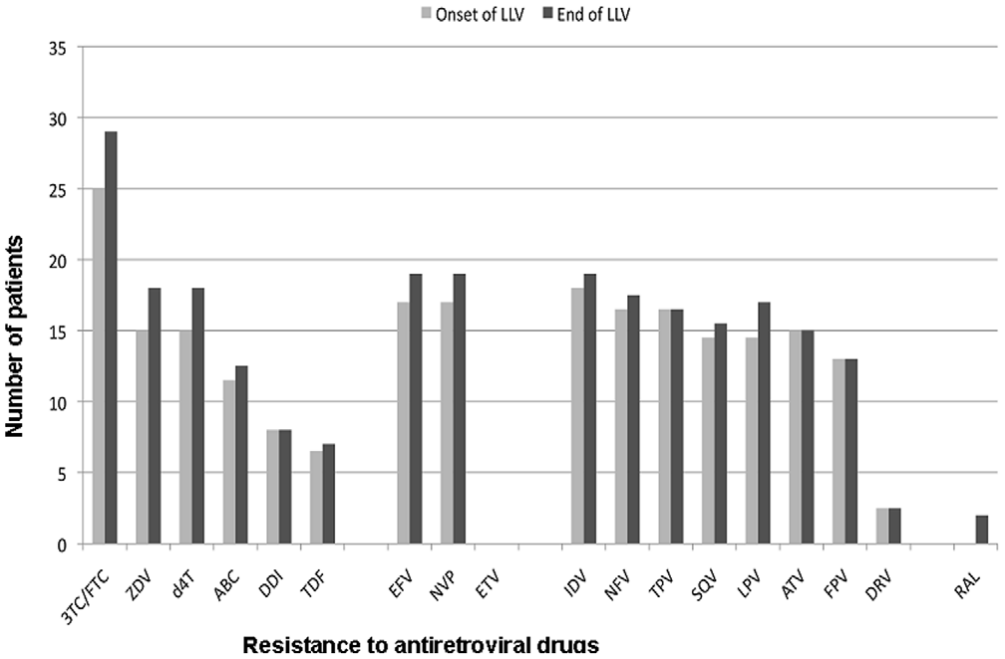
Resistance to antiretroviral drugs at the onset and the end of the low-level viremia period, assessed using the 2009 ANRS HIV-1 drug-resistance algorithm v18. LLV: low level viremia, 3TC/FTC: lamivudine/emtricitabine, ZDV: zidovudine, d4T: stavudine, ABC: abacavir, ddI: didanosine, TDF: tenofovir, EFV: efavirenz, NVP: nevirapine, ETV: etravirine, IDV: indinavir, NFV: nelfinavir, TPV: tipranavir, SQV: saquinavir, LPV: lopinavir, ATV: atazanavir, FPV: fosamprenavir, DRV: darunavir, RAL: raltegravir.

According to the 2009 ANRS HIV-1 drug-resistance algorithm v18, DRAM present in G1 or in previous RGT conferred resistance to a median (IQR) of 4.5 (1–11.9) antiretroviral drugs: 1 (0–4.4) for NRTI, 0 (0–2) for NNRTI and 1.75 (0.5–6) for PI (Figure 2). The median (IQR) GSS to the current ART was 3 (2–3).

### Acquisition of resistance mutations during LLV

Overall, 11 patients among 37, (30%), all pretreated, acquired at least one new DRAM (median 2, ranging from 1 to 9) over the study period (Table 2, Figure 1). New resistance mutations were observed for NRTI in 6 patients (16%), for NNRTI in 1 patient (2.7%), for PI (major mutations) in 4 patients (11%) and for RAL in 2 patients (5.4%). According to the current regimen, 3 patients from the 33 who received 3TC or FTC acquired M184V mutation (9%), one patient from the 8 who received NNRTI acquired K103N mutation (12.5 %), 4 patients from the 32 who received PI acquired major PI mutations (12.5 %) and 2 patients from the 7 who received RAL acquired IN mutations (29%).

During the LLV period, the median number of drugs with genotypic resistance increased from 4.5 at G1 to 6 (1.1–11.9) at G2 (Figure 2). The sensitivity analysis to antiretroviral drugs before and after the LLV period showed an increased resistance for 12 of the 18 studied drugs. The median (IQR) GSS to the current treatment remained 3 (2–4).

We investigated if some virological or therapeutic factors were predictive to the emergence of new DRAM during LLV. Among ART type, treatment duration, nadir and current CD4 cell count, baseline pVL, number of DRAM at G1, GSS, duration of LLV period, level of pVL during LLV and number of LLV episodes above 50 c/mL, no factors was significantly associated with the emergence of new DRAM (data not shown).

**Table 2:** Characteristics of the 11 patients in whom new resistance-associated mutations (RAM) were detected during low-level viremia.

Patient #	ART regimen	CD4 count at the onset of LLV (cell/mm^3^)	N. of previous genotypes	Baseline DRAM*	New RAM during low-level viremia	LLV duration (months)	pVL at time of RAM detection (copies/mL)	Months of treatment when RAM was detected
1	TDF/FTC+FPV/r	148	1	RT:L100I, Y188C	RT: M184I	10	257	21
				PR:K20M, M36I, L63P, V77I, L90M				
10	d4T = 3TC+ATV/r	396	0	RT:D67N, K70R, M184V	RT: K219E	9	88	40
				PR:L10I, K20R, M36I, M46I, I54M, L63P, A71T, V77I, I84V				
20	ABC/3TC+ATV/r	361	0	RT:D67N, K70R, M184V, K219E	RT:L74V	12	464	21
				PR:K20R, D30N, M36I, I50L, A71T	PR:L10F, L33F, I53L, L63P			
21	ddI+3TC+FPV/r	349	0		RT: M184V	7	256	15
				PR: K20R, M36I				
25	TDF/FTC+DRV/r+T20+RAL	136	6	RT:M41L, D67N, Y181C, M184V, L210W, K215Y		11	112	18
				PR:L10I, L33F, M36I, M46L, G48V, I54V, L63P, A71I, V77I, V82A, L90M	PR:I47V			
					IN: T97A, N155H			
30	TDF/FTC+ATV/r	545	2	RT:M41L, D69N, M184V, G190S		17	114	30
				PR: K20R, M46I, L90M	PR: I47V			
34	TDF+ABC/3TC+DRV/r+T20	232	4	RT:M41L, D67N, T69D, K70R, L74V, Y181C, M184V, G190A, T215Y, K219E		6	283	17
				PR:L10I, K20R, M36I, M46I, I84V	PR: L76V			
35	TDF/FTC+ETV+T20+RAL	249	2	RT: L74V, L100I, K103N, Y115F, M184V, T215Y		10	104	17
				PR:L10I, K20R, V32I, M46L, I47V, I53L, I54V, L63P, A71V, G73S, V82A, L90M				
					IN: T97A, Y143C, G163R			
36	ABC+ddI+EFV+ATV/r	497	0		RT: L103N, M184V, T215Y	11	152	43
					PR: G48V, L63P, A71V, V77I, V82T, I84V			
39	FTC+LPV/r+ATV+T20	29	0	RT: M41L, T69D, L74V, L100I, K103N, M184V	RT: D67N, L210W, T215Y	6	58	12
				PR: L63P				
45	TDF/FTC+DRV/r	386	1	PR: L63P, V77I	PR: L10I, L33V	13	81	24

* Baseline DRAM cumulated mutations detected in the genotype (G1) at the onset of LLV period and in all previous available genotypes.

DRAM: drug-resistance associated mutations, 3TC/FTC: lamivudine/emtricitabine, d4T: stavudine, ABC: abacavir, ddI: didanosine, TDF: tenofovir, EFV: efavirenz, ETV: etravirine, LPV: lopinavir, ATV: atazanavir, FPV: fosamprenavir, DRV: darunavir, RAL: raltegravir, T20: enfuvirtide, /r: ritonavir-boosted protease inhibitor, RT: reverse transcriptase, PR: protease, IN: integrase.

## Discussion

We retrospectively analyzed 48 patients from two hospital cohorts presenting LLV episodes defined by at least three pVL between 50 and 500 c/mL after 6 months of ART regardless of treatment lines and regimen. All these patients were followed in “real-life” conditions and were heterogeneous for ART history, for prior treatment failure and for presence of drug-resistant viruses. When comparing RGT before and at end of the LLV period, we found that new DRAM occurred in 30% of evaluable cases during LLV and according to the regimen, new mutations conferring resistance to NRTI, NNRTI, PI and raltegravir were observed in 14%, 12.5%, 12.5% and 29% of patients, respectively.

Detection, but not selection, of DRAM for pVL below 500 c/mL under ART, is frequent in transversal cohorts [14], [17] and a recent analysis using a simulation technique, showed that the risk for emerging resistance was estimated to occur in 65% during detectable VL, excluding blips, below 500 c/mL [17]. Our longitudinal analysis of genotypic drug resistance evolution during LLV showed that selection of DRAM could occur, even at low levels of viral replication (≤200 c/mL) and in patients receiving a PI/r containing regimen.

Ritonavir boosted PI-based regimen are at lower risk of selection of DRAM both in the RT and in the protease genes [18], [19]. We reported here the selection of new major DRAM in the protease gene in 4 patients receiving a boosted PI-based regimen (12.5%), when emerging PI resistance was rarely described during LLV [20]. In addition, we detected selection of TAM in also 3 patients. Duration of the LLV period of our study was long (median 11 months, up to 43 months), which could have contributed to the emergence of resistance to higher genetic barrier drugs such as tenofovir, zidovudine or ritonavir-boosted PI. We can not however exclude that these resistance mutations had been selected during previous VF, still present at minority level and/or archived in cellular reservoir and re-emerged upon a modification of antiretroviral pressure. Indeed, the selection of additional DRAM in our study, was observed only in pre-treated patients.

Emerging resistance to low-genetic barrier drug was detected in three patients for 3TC/FTC (9%) and in one for EFV (12.5%). Selection of DRAM during LLV was previously reported in 67% of naïve and pretreated patients receiving 3TC and EFV based-regimen [21], when in this study the median pVL during LLV was higher (417 c/mL). Furthermore in a sub analysis of two large trials in patients receiving an initial NRTI with EFV or LPV/r, or EFV and LPV/r containing ART, new DRAM were selected during LLV (median pVL of 77 c/mL) in 37% patients mainly at codons 184 and 103 of the RT gene but not in the PR gene [22].

In addition, new raltegravir-associated DRAM were detected in 2 of the 7 (29%) patients who were receiving raltegravir. Selection of DRAM in the integrase gene was previously reported in 8% of 39 highly-pretreated patients receiving a raltegravir and boosted-PI containing regimen, who experienced at least one episode of detectable viremia below 500 c/mL [23], but not confirmed elsewhere [24], [25]. Emerging resistance to raltegravir, otherwise considered to be a low-genetic barrier drug, seems to rarely occur during LLV in pretreated patients, suggesting a reduced fitness of highly mutated resistant viruses.

Finally, higher level of pVL and non-Hispanic black ethnicity were previously associated with the risk of emerging resistance in patients receiving an initial ART and presenting LLV [22]. Here we failed to identify risk factors associated to the acquisition of new DRAM, probably because of an unpowered sample size of patients retrospectively analyzed.

In conclusion, our study lead in standard clinical practice, confirms that new DRAM during LLV can emerge, regardless of the ARV antiretroviral target genes, and can remarkably reduce the current therapeutic options for further regimen. Both French and American 2010 antiretroviral guidelines recommend a rapid therapeutic intervention when VF if pVL is over 200 c/mL [6], [15], but a simple monitoring of pVL, drug-resistance and CD4 cell count evolution if pVL is below 200 c/mL. Our data underline here that persistent LLV is not a neutral virological circumstance in term of emergence of drug resistance, and for which RGT has a potential interest, in order to help to an early therapeutic optimization, whose efficacy still needs to be assessed in the future.
